# Informal Caregiver Burnout? Development of a Theoretical Framework to Understand the Impact of Caregiving

**DOI:** 10.3389/fpsyg.2019.01748

**Published:** 2019-07-31

**Authors:** Pierre Gérain, Emmanuelle Zech

**Affiliations:** ^1^National Fund for Scientific Research, Brussels, Belgium; ^2^Person Centred Research and Training Lab, Psychological Sciences Research Institute, Université catholique de Louvain, Louvain-la-Neuve, Belgium

**Keywords:** informal caregiver, family caregiver, burnout, burden, carer, exhaustion

## Abstract

Informal caregiving is a rewarding but demanding role. The present theoretical framework proposes to adapt the tridimensional concept of burnout to informal caregiving as a way to address the potential consequences of caregiving. This adaptation reflects caregivers’ reported difficulties, as well as empirical findings on emotional exhaustion, depersonalization, and personal accomplishment as caregiving outcomes. But to understand burnout in informal caregiving contexts, it is also necessary to find ways to model it. The Informal Caregiving Integrative Model (ICIM) is thus proposed. This model is based on the integration of elements from literature on both informal caregiving stress and professional burnout. The goal of the ICIM is to emphasize the importance of every category of determinants of informal caregiver burnout (i.e., relating to the caregiver, the caregiving setting, and the sociocultural context), with a key mediating role for the caregivers’ appraisal of their situation and their relationship with the care-recipient. This article is a first integrative step in the consideration of a form of burnout specific to informal caregivers and supports the design of empirical and interventional studies based on the theoretical foundation that the ICIM proposes.

## Introduction

### Informal Caregivers

Informal caregivers are individuals voluntarily caring for a relative or a friend facing illness, disability, or any condition requiring particular attention (Schulz and Tompkins, 2010). Across the literature and in the media, it is widely accepted that being an informal caregiver puts a person at risk of poorer mental (Pottie et al., 2014; Sallim et al., 2015) and physical health (Kiecolt-Glaser et al., 2003; Känel et al., 2006; Capistrant et al., 2012). Although this consensus is contested (Brown and Brown, 2014; Roth et al., 2015), it nevertheless appears that being an informal caregiver can represent, in many ways, an experience that puts the individual under stress (Revenson et al., 2016).

The present work first proposes to adapt the concept of burnout to the context of informal caregiving as a means of addressing the consequences of such stress. As will be discussed, this adaptation is in response to concerns expressed in the literature about the measurement of the caregiving impact, currently driven by the study of the *subjective burden*. To drive future research on informal caregiver burnout, a new conceptualization must be constructed. To do so, two major models of caregiving stress and occupational burnout will be reviewed to identify their strengths and limitations. The Informal Caregiving Integrative Model (ICIM) will then be presented. The ICIM is designed to capture what makes existing models of professional burnout and informal caregiving stress so valuable, while trying to address their potential weaknesses.

### Informal Caregivers’ Burnout

Burnout is a tridimensional syndrome in response to chronic stress (Maslach et al., 1996). The concept is mainly used in the study of well-being at work, but it is interesting to note that one of the clinical observations at the origin of its conceptualization was not based on workers, but on volunteers at a support center (Freudenberger, 1974). This was a first step in considering that burnout could occur outside the occupational context (Schaufeli and Taris, 2005). This observation later led to the consideration of burnout in other settings, such as among parents (Mikolajczak et al., 2018), or students (Gustafsson et al., 2017).

The first reference to burnout in informal care was made in 1986. A study drew attention to “Spouse Burnout Syndrome,” because some spouses of patients with chronic diseases showed symptoms comparable to those experienced by formal caregivers in burnout (Ekberg et al., 1986). Despite its promising premises, this work received little attention for a long time. Since the first decade of the new millennium, however, an increasing number of studies have adapted burnout measures to assess informal caregivers’ strain. In line with Ekberg’s study in 1986, these studies have highlighted that some informal caregivers face strains comparable to the experiences of professionals in burnout (Angermeyer et al., 2006; Perkins and Hewitt, 2016; Thorson-Olesen et al., 2018).

In this view, caregiver burnout can be defined as a tridimensional syndrome in response to the stress that the caregiving context may represent. Emotional exhaustion can be defined as a feeling of overload, of no longer being able to continue, of being emotionally drained when facing the caregiving situation and the care-recipient (Thompson et al., 2014; Goodwin et al., 2017). Depersonalization describes the detached response in the relationship to the person being cared for. Taken to the extreme, this can go as far as the reification of the latter. Personal accomplishment encompasses the positive dimension of the helping experience. This dimension of personal achievement goes beyond the notion of evaluation by highlighting that the caregiver may gain a sense of fulfillment through his or her care work and find meaning in it (Cross et al., 2018). In the context of burnout, this positive sense of accomplishment tends to be reduced.

This tridimensional transposition must, of course, be empirically investigated. Although this is only an indirect indicator, studies that have transposed burnout measures into the informal help context do not seem to have any major psychometric problem, and the internal consistency indicators seem comparable to those found in the literature on professional burnout (e.g., Truzzi et al., 2012; Akinci and Pinar, 2014; Katsifaraki and Wood, 2014; Yan, 2014). In the literature, emotional exhaustion is often highlighted in studies of informal caregivers under the heading of general exhaustion. This exhaustion can occur at both physical and mental levels, but remains primarily emotional in nature (Galiatsatos et al., 2017). There is little evidence of cases of depersonalization in informal caring contexts, except for studies adapting the Maslach Burnout Inventory, the most frequently used burnout scale. Other studies have shown that some caregivers put an emotional and psychological distance between themselves and the person they are caring for in order to preserve themselves (Cross et al., 2018). This distance can take the form of a more pragmatic and distant style of care and relationship in the face of significant stress (Hubbell and Hubbell, 2002). Personal accomplishment has been investigated in terms of personal growth resulting from the caregiving role, positive impact on the care-recipient, and caregivers’ sense of acting in accordance with their values (Cross et al., 2018). Although the positive and negative impacts of informal care are related, the positive impact may be relatively unaffected by the negative caregiving experience and flourish independently (Lawton et al., 1991; Appleton et al., 2018). All these elements suggest that informal caregiver burnout is a promising concept to assess the impact of caregiving.

### The Burden-Burnout Relationship

Despite this promising position, the literature studying the negative impact of informal care remains focused on the concept of subjective burden. Subjective burden is the subjective assessment of the stress that the helping situation can represent (Galiatsatos et al., 2017). It is the caregiver’s appraisal of the objective experience (Lawton et al., 1991). Despite this seemingly clear definition, the concept of burden has been strongly criticized, with researchers pointing out that burden remains poorly defined and its assessment too vague (Mosquera et al., 2016). Depending on the definition used by authors, subjective burden may refer to the physical, psychological, emotional, social, and/or financial consequences of caregiving. This conceptual heterogeneity leads to diverse forms of assessment (Van Durme et al., 2012), and renders its use in public policy or research too ambiguous (Bastawrous, 2013). A concept defined too heterogeneously makes it difficult to draw clear conclusions. Contributing to this confusion, authors use the notions of subjective burden and caregiver burnout without distinction by measuring burden and reporting that they have measured burnout (and vice versa) (e.g., Schoenmakers et al., 2009; Kokurcan et al., 2015; Ghane et al., 2016).

The present work reflects the suggestion in the literature that subjective burden should be considered as the subjective experience of the caregiver, their perception of their caregiving role (Zarit and Zarit, 2015). Thus, subjective burden can be seen as an appraisal, an evaluation of how much the situation represents a source of stress for the individual, taking their resources into account (Lawton et al., 1991). This appraisal reflects the primary and secondary evaluations in Lazarus and Folkman’s stress theory (Lazarus and Folkman, 1984). The use of the appraisal, and of Lazarus and Folkman’s theory, is often a common basis for the different informal caregiver stress models (Schulz and Sherwood, 2008). The subjective burden thus appears as a key mediator between the demands of caregiving and the caregiving outcomes, such as informal caregiver burnout (Revenson et al., 2016).

### Understanding Caregiver Burnout: Existing Models

Studying informal caregiver burnout requires a theoretical basis on which to build an understanding of the burnout process. Existing studies addressing informal caregiver burnout seem to do so without this theoretical basis. The aim of the present work is thus to propose a theoretical model to guide future research on informal caregiver burnout. Such a model will need to combine elements of existing models from the literatures on caregiving stress and occupational burnout. In informal caregiving, the adaptation of Lazarus and Folkman’s stress model has been preponderant in most research; by contrast, various models have been investigated in occupational burnout, although over the past decade, the Job Demands-Resources (JD-R) Model has provided a clear framework for research (Schaufeli and Taris, 2014).

#### Model of Carer Stress and Burden

Several researchers have conceptualized and evaluated models to understand how caregiving stress occurs and affects the individual. Two models have been particularly investigated. The first one of these, which has been widely used, is the stress process model (Pearlin et al., 1990), and the second is the appraisal model (Lawton et al., 1991). A combination of these two models was proposed by Sörensen et al. (2006) in the Model of Carer Stress and Burden, an integrative model of the caregiver stress in the case of neurodegenerative disease.

**Figure 1:**
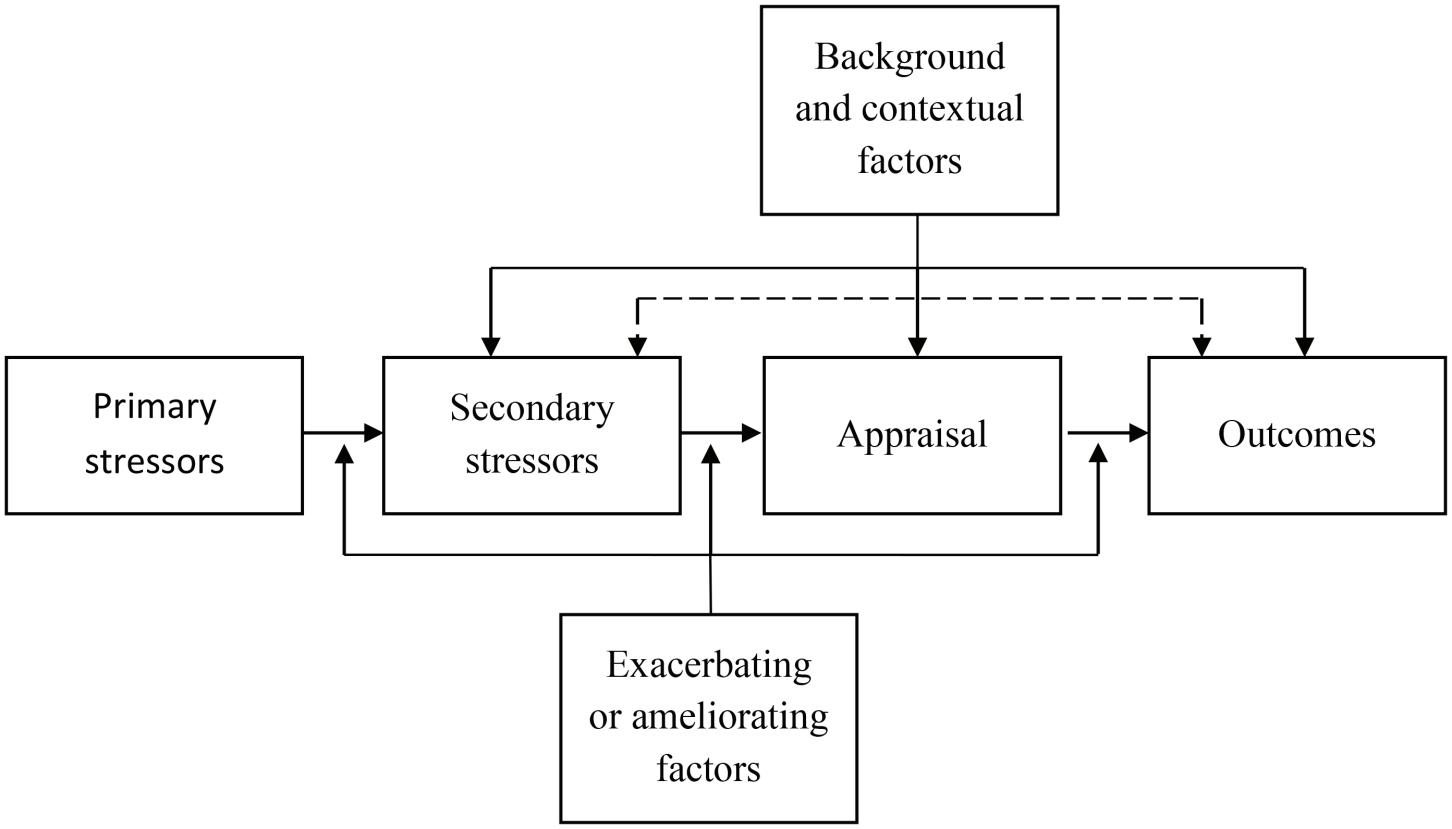
Model of Carer Stress And Burden [adapted from Sörensen et al., 2006 Copyright (2006), with permission from Elsevier].

The model breaks down the process into six different interacting elements (see Figure 1). (1) Primary stressors are all objective elements in the caregiving setting, such as the type and intensity of symptoms, the tasks to perform or the intensity (hours/week spent caregiving). These primary stressors cause (2) secondary stressors, the consequences of the objective elements (e.g., lack of free time, family conflicts, financial strain). These are the mediators between the primary stressors and (3) the appraisal. The appraisal is the caregivers’ subjective assessment of their situation. According to Lazarus and Folkman’s theory (1984), this is an evaluation of the equilibrium between demands and resources. This evaluation leads to (4) the outcomes. These outcomes are psychosocial (e.g., depression or well-being), but could also be behavioral (e.g., substance consumption), or physiological (e.g., health issues related to chronic stress). This linear succession is influenced by (5) exacerbating and mitigating factors. These are all the elements other than primary and secondary stressors that modify the relationships between the variables. Coping strategies, personality facets, and other resources are among the factors modifying the relationships between primary and secondary stressors, appraisal, and outcomes. Finally, (6) background and contextual factors such as sociodemographic and cultural or ethnic determinants frame the caregiver’s experience.

The Carer Stress and Burden Model and the two other models it takes its origin from make a crucial distinction between primary and secondary stressors, acknowledging the distinct role of primary stressors and their consequences (Pearlin et al., 1990). They also emphasize the central mediator role of appraisal between objective stressors and outcomes (Lawton et al., 1991). However, this model only focuses on caregiving stress. Caregivers’ psychological and social determinants are considered peripheral, although they play an important role in their appraisal and experience of caregiving strain (Adelman et al., 2014). The dyad is not considered, neglecting the relation between caregiving strain and the relationship with the care-recipient (Spruytte et al., 2002; Kindt et al., 2015). The appraisal is only defined by secondary stressors and background elements, yet primary stressors, the relationship with the recipient, and the caregiver’s individual characteristics may also contribute to this appraisal (Cuijpers and Stam, 2000). Dispositional and situational coping strategies are not explicitly integrated in the model, even though it is based on Lazarus and Folkman’s work, and despite the crucial role played by coping strategies in the caregiving stress regulation (Pinquart and Sörensen, 2005). Subjective burden is considered as an outcome and not an appraisal. Finally, there are few feedback loops, suggesting that all of the caregiving stress model elements lead to outcomes without any impact of these outcomes on caregiving in return.

#### The Job Demands-Resources Model

**Figure 2:**
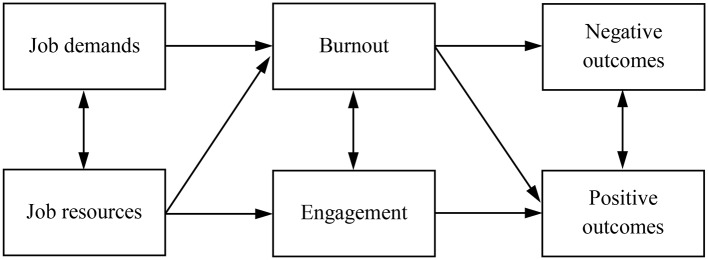
The Job Demands-Resources Model [adapted by permission from Springer Nature, Springer ebook by Schaufeli and Taris, 2014].

In the burnout literature, one model synthesizes the way burnout appears in occupational contexts: the Job Demands-Resources Model (Demerouti et al., 2001). This model presents burnout as a two-dimensional process. On the one hand there is exhaustion, the wearing down of levels of energy, and the depletion of the caregiver’s emotional resources. On the other hand there is engagement in the job, the willingness to perform well and to find new positive and constructive challenges within the work. In this view, burnout is caused by demands (stressors) and is diminished by resources (see Figure 2).

This model presents the role of burnout as a mediator between demands/resources and outcomes: the stressors (and resources) have a direct impact on burnout, which in turn causes outcomes. It also makes it clear that negative processes (e.g., burnout) do not obliterate positive ones (Broese van Groenou et al., 2013). Although this has not been assessed directly (except for parental burnout: see Mikolajczak and Roskam, 2018), the model postulates an equilibrium between demands and resources. It is the imbalance between persistent demands and insufficient resources that will, in the end, lead to burnout. Although this balance is a core element in the JD-R, it is not directly incorporated in the model, and direct effects of demands and resources are emphasized instead. However, this balance could be thought of as similar to Lazarus and Folkman’s appraisal, which is explicitly included in caregiving stress models.

The conceptual difference between demands and resources is questionable (Schaufeli and Taris, 2014). The rationale is that demands are negatively appraised while resources are positively appraised and contribute to a more positive experience. However, meta-analytic studies have shown that using a dual set of positive and negative determinants is simplistic and reductive (Crawford et al., 2010), and this has led to a multiplication of versions of the model (Bakker and Demerouti, 2017). Moreover, the conceptual difference between low demands and high resources (and vice versa) can sometimes be subtle. If the (im)balance between demands and resources is crucial to understanding the experience of caregivers, a clear distinction between the two may thus prove less useful.

### The Informal Caregiving Integrative Model

The consideration of both the caregiver stress model and the JD-R model has made it possible to identify important factors to consider when building an understanding of informal caregiver burnout. Such a conceptualization should: (1) consider stressors and resources not only in the caregiving setting but also in the caregiver’s psychosocial characteristics, (2) take into account the relationship with the care-recipient as a critical component in the understanding of the caregiving experience, (3) consider burnout as a key mediator between stressors and outcomes, (4) integrate the caregiver’s appraisal as a core element in the model, (5) consider subjective burden as a measure of appraisal, and (6) include feedback loops. In addition, the consideration of determinants of caregiving should not focus on the often arbitrary distinction between demands and resources, but rather aim at understanding the processes by which these determinants may impact the caregiver’s strain and appraisal.

**Figure 3:**
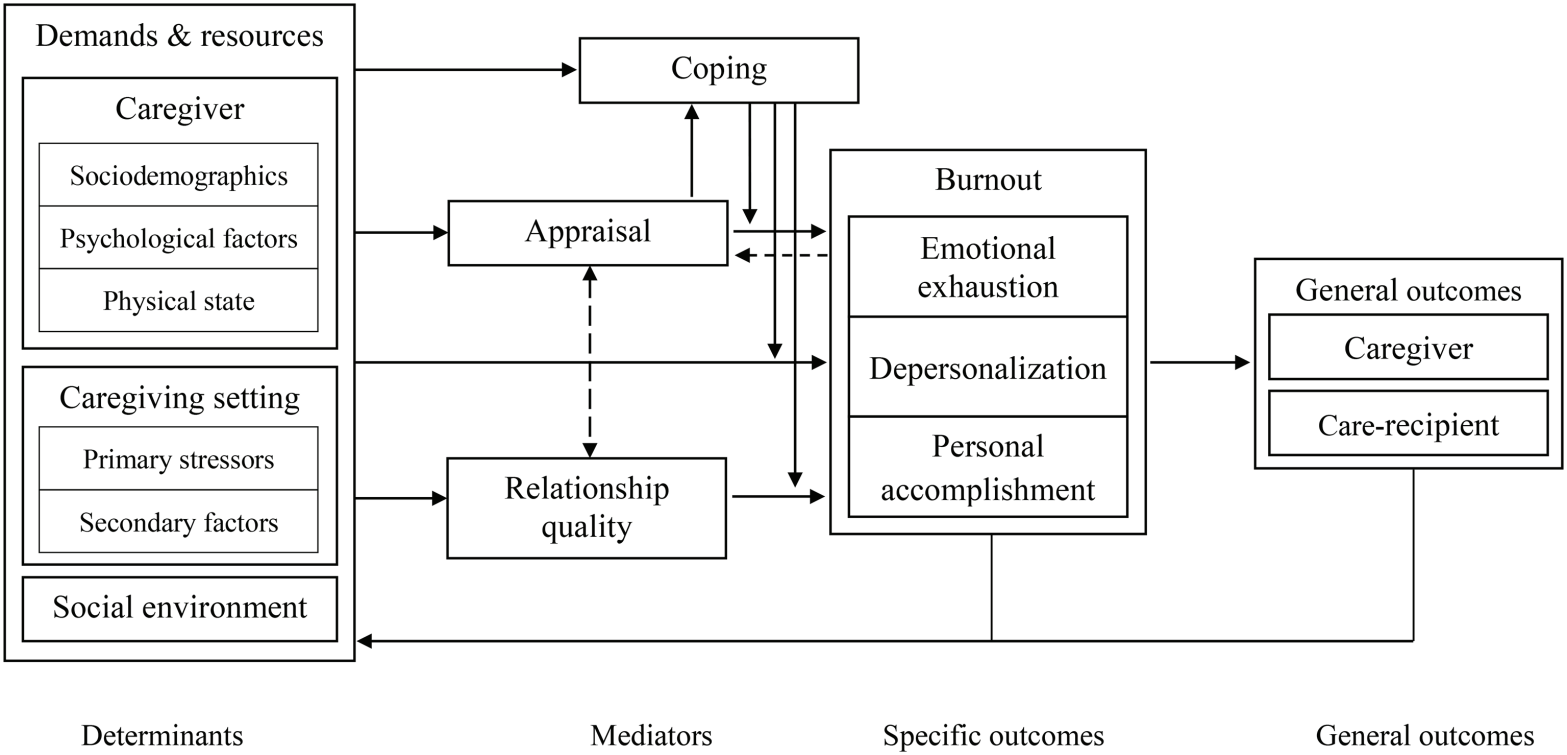
The Informal Caregiving Integrative Model (ICIM).

To respond to these requirements, the Informal Caregiving Integrative Model (ICIM) is proposed as a theoretical framework to guide future research (see Figure 3). The rationale of the ICIM is to consider the different determinants of informal caregiver burnout (i.e., the caregiving setting, the caregiver, and the environment) on the same footing. Burnout is conceived of as a key mediator between these determinants and general outcomes, and the impact of the determinants on informal caregiver burnout is mediated by both the caregiver’s appraisal and his or her relationship quality with the care-recipient.

To date, several elements of the model have already been explored by studies that focus on informal caregiver burnout. Because the ICIM is a theoretical model aiming at framing future research, the elements described in each section of the model do not aim at being exhaustive. The present goal is rather to illustrate the model with existing studies, mainly on informal caregiver burnout, and to suggest future directions for research in this field.

#### Determinants: Caregiver’s Characteristics

The first set of determinants of caregiver burnout are the caregiver’s characteristics. Caregivers remain individuals with personal cognitions, emotions, and motives that should be considered when aiming at understanding informal caregiver burnout. The characteristics of the caregiver can be divided into three main groups: background and sociodemographic, psychological, and physical factors.

##### Background and Sociodemographic Factors

The caregiver faces several elements that cannot be changed but still influence his or her caregiving strain. Being a female caregiver has been found to be associated with a heavier burden (Salama and El-Soud, 2012) and more burnout (Angermeyer et al., 2006; Truzzi et al., 2012). Often, the effect of gender has been confused with the effect of other gendered issues such as caregiving being more frequent among women than men (Revenson et al., 2016), and this has led to other studies finding no relation between gender and caregiver burnout (Kokurcan et al., 2015; Onwumere et al., 2017). Regarding the caregiver’s age, no stable effect has been found (Demirhan et al., 2011; Truzzi et al., 2012; Yan, 2014; Onwumere et al., 2017). However, a difference may lie in the type of relationship with the recipient, reflecting that assuming the caregiving role has different implications at different life stages (Broese van Groenou et al., 2013; Perkins and Hewitt, 2016).

Still working while providing informal care may represent a protective factor in the sense that it relieves the caregiving strain by providing an emotional distraction, even if it is likely to exposes to work-related stressors (as suggested by Kokurcan et al., 2015). This protective effect of working may, however, be biased since caregivers sometimes take early retirement or a career break to provide care. The remaining working caregivers would thus be the ones with less caregiving responsibilities and less caregiving stress (Williams et al., 2016). In a similar way, it is important to note that keeping working is often related to gender disparity: women are more likely to become caregivers than men, and to reduce their working time to do so (Verbakel et al., 2017). Regardless of the cause, the impact of professional status remains entangled with financial matters and caregivers experiencing financial strain are more prone to subjective burden and burnout (Lindström et al., 2011; Chiao et al., 2015; Götze et al., 2015). In light of all these elements, the impact of the professional status is therefore much more complex than it may seem.

Research on role theory has suggested the significant impact of the caregivers’ multiple roles (Bastawrous, 2013). The accumulation of roles, such as being a parent at the same time as being an informal caregiver, also adds to the caregiver’s strain (Stephens et al., 2001; de Almeida Mello et al., 2016). Recent studies have shown such patterns in professional burnout. Double-duty caregivers – being a formal and informal caregiver at the same time – are at higher risk of professional burnout than work-only caregivers (Häusler et al., 2017; DePasquale et al., 2018). In the same way, being an informal caregiver has been shown to increase the chances of parental burnout (Lindström et al., 2011; Gérain and Zech, 2018; Séjourné et al., 2018). The informal caregiving role thus seems to affect the caregiver’s other roles. This permeability is probably bidirectional, and the impact of the other roles on caregiving strain must be investigated to fully understand what is at stake in caregiver burnout.

As with gender or professional status, the relevance of studying background or sociodemographics lies not in the variable itself but rather in what it implies. The impact of gender would rather reveal gender discrimination, professional status may represent a resource or a demand, and role accumulation can be a major risk factor for multiple aspects of life. Future studies integrating these determinants should therefore investigate their meaning rather than the variable itself.

##### Psychological Factors

Many psychological factors have been investigated among informal caregivers. Individuals experience a diverse set of emotions when caregiving, related to the relationship with the recipient, the gravity of the recipient’s condition, the caregiving role, and their own life. Emotion regulation is thus particularly essential to them and plays an important role in their caregiving experience. One study highlighted that alexithymia was a risk factor for burnout, especially for emotional exhaustion and depersonalization (Katsifaraki and Wood, 2014). Similarly, emotional competencies appear to be a promising resource for the caregiver. Emotional competencies have been found to play a role in preventing professional and parental burnout (Görgens-Ekermans and Brand, 2012; Mikolajczak et al., 2018) and identified as a promising target of intervention to reduce informal caregivers’ psychological distress (Weaving et al., 2014). More broadly, personality traits also seem to have an impact on subjective burden and informal caregiver burnout (Chiao et al., 2015; Gérain and Zech, 2018).

One of the ways to consider emotion regulation is in terms of individuals’ ways of coping. Caregivers who have a submissive or helpless approach (Duygun and Sezgin, 2003; Yılmaz et al., 2009) or who engage in denial (Onwumere et al., 2017) appear more prone to caregiver burnout than caregivers with coping strategies such as confident and optimistic approaches (Yılmaz et al., 2009) or positive reappraisal and active coping (Onwumere et al., 2017). More generally, the use of a wider range of coping strategies appears to lead to less subjective burden (Adelman et al., 2014). However, these coping styles are global dispositions regarding emotion regulation. The investigation of the coping strategies used when actually experiencing the situation – e.g., through ecological momentary assessment – would allow us to understand if they are effective responses to the stress caregivers face.

Cognitions, especially (dys)functional thoughts and perceived competence, also play an important role in the caregiving experience. Perceived ability to cope with the care-recipient’s illness or behaviors is a predictor of burden and burnout (Cuijpers and Stam, 2000). It is directly related to caregivers’ needs for knowledge regarding the recipient’s health issue (Zarit and Zarit, 2015), and to self-efficacy (Ducharme et al., 2011).

Regarding the caregiving role, lack of choice in becoming the caregiver is associated with higher subjective burden (Adelman et al., 2014). Intrinsic motivation to care appears to be a protective factor for informal caregiver burnout (at least for emotional exhaustion, in Kindt et al., 2015). Constant worrying and need for control in the caregiving role appear to be risk factors, as they require chronic alertness on the part of the caregiver (Cuijpers and Stam, 2000; Lindström et al., 2011). More generally, a strong sense of coherence appears to be a protective factor against burnout (Goetzmann et al., 2012; Götze et al., 2015).

All these studies point to the necessity to consider psychological factors when studying caregiver burnout. While some of them are more related to trait elements (e.g., personality) that would be hard to modify, others could be targeted by interventions to help exhausted caregivers or to prevent future problems. Future research should thus fully identify the psychological processes involved in informal caregiver burnout in order to identify those with the greatest impact and those which are most promising for interventions.

##### Physical State

The physical health of caregivers is a factor that determines their involvement in caregiving. Healthy caregivers often take more responsibility in caregiving (Pinquart and Sörensen, 2007). The experience of somatic disorders, illness, or chronic pain has been shown to put pressure on caregivers, making them more prone to burnout (Hattori et al., 2000, 2001; Demirhan et al., 2011). While this is especially acute for aging caregivers, it is a factor in all caregivers’ capacities to provide care and in the difficulties they may face. Future research should thus consider more closely how the evolution of caregivers’ physical state could affect their ability to manage the situation and impact their mental health.

#### Determinants: Caregiving Setting

As pointed out in the Sörensen’s Model of Carer Stress and Burden, a distinction needs to be made between primary and secondary stressors in the caregiving setting. Primary stressors are all the demands (and potential resources) *defining* the caregiving role. They are mostly referred to in the literature as “objective burden” (Brouwer et al., 2004). Secondary stressors are all the demands and resources *caused by* the primary stressors.

##### Primary Stressors

Time since start of caregiving (duration) is often considered as a central element in caregiving strain. Yet it appears that there is no relationship between duration of care and burnout (Sugihara et al., 2004; Lindström et al., 2011; Kokurcan et al., 2015), and only a weak link to burden (Pinquart and Sörensen, 2003; Adelman et al., 2014). A similar pattern appears regarding time spent caregiving per week (intensity) (Angermeyer et al., 2006; Truzzi et al., 2012; Adelman et al., 2014; Kokurcan et al., 2015), and living with the care-recipient has also been highlighted as an inconclusive stressor for subjective burden and burnout (Cuijpers and Stam, 2000; Cheung and Chow, 2011; Yan, 2014; Chiao et al., 2015).

Being a spouse appears to be a risk factor (Cuijpers and Stam, 2000; Sugihara et al., 2004), and caring for a man appears to be more demanding than for a woman (Goetzmann et al., 2012; Onwumere et al., 2017). The reason behind the increased risk of caring for a man has not been investigated yet, but the low effect size of this association suggests that intensive investigation is not required.

Informal caregivers provide care to individuals with a wide diversity of health issues. Most studies, however, consider informal caregivers in the context of one particular health issue (e.g., neurodegenerative diseases, chronic pain, or disability) in order to control for a significant form of variability. Some studies have compared different populations in their sample (often one by one) (e.g., Weiss, 2002; Ybema et al., 2002; Lindström et al., 2010; Tramonti et al., 2019), but not enough to conclude about a particular risk profile. The autonomy (or functional impairment) of the care-recipient is a factor often related to caregiving subjective burden and burnout (Cheung and Chow, 2011; Yan, 2014; Chiao et al., 2015; Lynch et al., 2018), but this variable remains specific to some populations (e.g., dementia). The common ground is the intensity of the symptoms in each health issue, which appears to be more related to caregiving subjective burden (Chiao et al., 2015). Such evidence has also been highlighted for informal caregiver burnout in caregivers of individuals with mental illness (Kokurcan et al., 2015) or dementia (Cheung and Chow, 2011; Truzzi et al., 2012; Yan, 2014), and in caregivers of children with disability (Demirhan et al., 2011; Basaran et al., 2013), chronic disease (Riva et al., 2014), or comorbid issues (Gérain and Zech, 2018). Beyond the health issue itself, correlative analyses have also shown a relationship between recipient’s well-being and depressive symptoms on the one hand and subjective burden and informal caregiver burnout on the other, which suggests a mutual influence of the emotional states in the dyad (Ybema et al., 2002; Yılmaz et al., 2009; Adelman et al., 2014; Kindt et al., 2015).

Primary stressors thus seem to have an impact on informal caregiver burnout. However, this impact appears mostly related to the health issue and not to descriptive characteristics of the caregiving role. Future research should therefore focus not only on the objective stressors, but also on what they entail (i.e., secondary stressors), and what they represent for the caregivers (i.e., their appraisal).

##### Secondary Stressors

Secondary stressors are often investigated less than primary stressors when exploring caregiving stress and its impact, yet studies have shown a significant role of these stressors in caregiving strain. Some have highlighted that informal caregivers reporting high levels of informal caregiver burnout perceived themselves as having a more disturbed daily life due to the illness, less free time and time for themselves (Lindström et al., 2011), or hypervigilance regarding the occurrence of new symptoms in the case of unpredictable illnesses (Lond and Williamson, 2017). Informal caregiver burnout was also found to be related to having a reduced social life and the loss of friends (Lindgren, 1990), and more globally the feeling on the part of caregivers of having had to give up important things for themselves due to the care-recipients’ problem (Gérain and Zech, 2018). Beyond the emotional strain, such feelings could also have an important impact on the relationship with the recipient, with a mixed feeling of duty and resentment toward the care-recipient (Williams et al., 2016).

Although it has not been widely investigated yet, the existence of secondary resources remains open to consideration. From this perspective, a resource arises because of the presence of a primary stressor, such as more social support or less isolation, due to caregivers’ support groups or an increase in regular social support (Sakakibara et al., 2015).

#### Determinants: Social Environment

The caregivers’ social environment can be considered in three distinct ways: informal and partner support, professional support, and the sociocultural environment. Informal social support appears to diminish burden (Choi and Sok, 2012; Adelman et al., 2014) and burnout (Choi and Sok, 2012; Riva et al., 2014; Kokurcan et al., 2015), but this protective role is more important when the support responds to a caregiver’s specific need (Lindström et al., 2011). Because caregivers often take sole responsibility for care, the presence of other informal caregivers may represent a resource (Peeters et al., 2010). However, such a resource could also translate into more stress due to additional conflicts, perceived inequities, worries, or diverging opinions (Williams et al., 2016). Outside the caregiving setting, the face-to-face or online support of other caregivers also seems to be beneficial for caregivers because they share comparable experiences (Perkins and Hewitt, 2016).

The partner is not always directly involved in caregiving but it appears that their support is essential to the provision of informal care (e.g., DePasquale et al., 2018). Research on support between parent-caregivers – parents providing care to one of their children – has shown that an unsatisfactory marital relationship and disagreements with the partner are related to burnout, highlighting the need for parents to face this challenge together (Lindström et al., 2011; Riva et al., 2014; Gérain and Zech, 2018). Besides parent-caregivers, it may also be interesting to consider the quality of the relationship with the partner in the context of caring for a relative, other than a child, to understand how the caregiving situation modifies the dynamic in the couple. The particular situation of being the partner’s informal caregiver will be addressed in the section on the relationship with the care-recipient.

Whether from the couple or the social network, the question of social support raises the broader and complex issue of caregivers’ isolation. This isolation may originate from the social environment or from the informal caregiver (Priestley and McPherson, 2016; Lindström et al., 2017). Whichever is the case, it affects the caregiver in an overall sense and could contribute to the caregiver’s burnout, whether directly or indirectly through his or her appraisal (Vasileiou et al., 2017).

Regarding professional support, one study has shown that difficult relationships with healthcare professionals are related to caregiver burnout (Almberg et al., 2000). There is no clear evidence of the impact of the use of support and respite services (Vandepitte et al., 2016). Certain in-home services seem to diminish the burden, but informal caregivers calling on such services who have waited a long time before doing so tend to be more exhausted than average (Sugihara et al., 2004). It is difficult to be sure how useful a resource it could represent for caregivers in general, especially given the potentially deleterious impact on the caregiver’s experience, due to the additional stress it might bring. Further research should focus on ways of improving support for informal caregivers and understanding the motives of informal caregivers who call on formal support.

The sociocultural environment of the individual is too often neglected in burnout research (Pines et al., 2011), and informal caregiver burnout is no exception. Culture seems to be an important factor in the consequences of informal caregiving (Pinquart and Sörensen, 2005; Chiao et al., 2015). At first, depending on their cultural norms, individuals could see their transition to the caregiving role as a normal process, or on the contrary experience it as a burden (Sutter et al., 2016). Later on, the perceived normality or abnormality of the caregiving tasks performed and the trade-off made due to caregiving may also impact their appraisal (Chiao et al., 2015; Konerding et al., 2018). As the care-recipient’s health issue progresses, an unwillingness to delegate tasks and receive assistance due to cultural reasons may lead to burnout in the caregiver (Scharlach et al., 2006). More broadly, there is a strong need to consider the cultural perspective and to be aware of the need to include groups often under-represented in research (Parveen et al., 2018). Future research focusing on cultural aspects and their implications in caregiving will lead to a more precise understanding of caregivers’ experience in different contexts and how it affects their well-being (Bastawrous, 2013). In light of existing studies, such future work should try to understand the mechanisms by which culture affects caregiver stress and burnout (e.g., Knight and Sayegh, 2010).

#### Caregiving Appraisal

In the Informal Caregiving Integrative Model, all the determinants are held to have a direct impact on the caregiving appraisal. Absent from the JD-R, this appraisal is the subjective evaluation of the caregiving experience by the caregivers themselves. It is the evaluation of the balance or imbalance between demands and resources and is thus the weighting of the determinants. This evaluation is a mixture of positive and negative assessments, but its most investigated aspect in research is the subjective burden (Galiatsatos et al., 2017). Close correlations have been found between subjective burden and emotional exhaustion, while less significant relationships have also been found with depersonalization and personal accomplishment (Kasuya et al., 2000; Angermeyer et al., 2006; Kyung-Bock and Kim, 2008; Truzzi et al., 2008; Özlü et al., 2009; Choi and Sok, 2012; Salama and El-Soud, 2012; Akinci and Pinar, 2014; Götze et al., 2015). These results suggest that the impact of the appraisal on outcomes could be mediated by burnout (Lee and Singh, 2010). Other appraisal elements such as feeling trapped in the caregiving role have not been investigated yet, but are probably related to caregiver burnout (Sörensen et al., 2006).

Future work should focus on a more homogeneous definition of what form a positive appraisal could take, as opposed to the long-standing focus on subjective burden. A positive appraisal appears to have a positive impact on caregiver well-being (e.g., Yamamoto-Mitani et al., 2004), but this has not been investigated in connection with informal caregiver burnout. Focusing on positive appraisal could also have the broader goal of moving away from the long-standing view of caregiving as an ultimately negative experience, to a more balanced view in which positive appraisal is at the core of the evaluation (Brown and Brown, 2014).

#### Relationship Quality

The relationship quality has been little considered in caregiving stress models, but the relationship with the recipient is the reason why an individual becomes a caregiver and remains a central element when considering either the care-recipient or the caregiver (Revenson et al., 2016). The occurrence of an illness or a disability modifies the roles and the relationship in the dyad between the future care-recipient and the future caregiver (Spruytte et al., 2002; Bastawrous et al., 2015). As the caring process progresses, the relationship quality remains a core-element in the caregiving experience. Of course, relationship quality with the care-recipient is determined by many elements, such as the relationship before caregiving or attachment style, but the key is to consider the impact caregiving may have on this relationship. Poor relationship quality has been found to be related to burden (Cuijpers and Stam, 2000) and burnout (Goetzmann et al., 2012; Kindt et al., 2015). Inequity in the relationship is also a key factor for caregivers of a spouse, particularly when the caregiving spouse feels like he or she is of minor importance in the couple, having been overshadowed by the other partner’s health issue (Ybema et al., 2002). One study has highlighted the need to take into account the disease’s temporal characteristics when exploring further the impact of this relationship (Fauth et al., 2012). Relational closeness appears to be a resource when the disease is manageable, and death remains a distant prospect. But when death appears closer, there may be a need to prepare for the separation.

Future research should focus on developing a deeper understanding of how the relationship quality could be a mediator between determinants and caregiver burnout. Dyadic coping has not been investigated in the context of informal caregiver burnout, but it appears to be a promising area for research in light of existing results (e.g., Rottmann et al., 2015). Investigation should also be broadened to the dyadic processes involved in caregiving, and to dyads other than couples (Revenson et al., 2016). It should also address the relationship between appraisal and relationship quality and its evolution over time. If positive relationships could alleviate part of the subjective burden (Lea Steadman et al., 2007), other elements such as perceived fairness or unfairness or modifications in roles could also impact the caregiver’s appraisal (e.g., McPherson et al., 2010).

#### Informal Caregiver Burnout

Informal caregiver burnout is the key element of the ICIM. It is expected to be the consequence of the different sets of determinants, either directly or through the mediation of the appraisal and the relationship quality with the care-recipient. Caregiver burnout is also viewed as a key mediator between demands and various more general outcomes, as highlighted in several studies (Lee and Singh, 2010; Kindt et al., 2015).

As pointed out in work on professional burnout, the negative impact of caregiving may overshadow the positive impact, but this does not mean that the latter cannot exist in presence of the former (Schaufeli and Taris, 2014). Positive and negative caregiving impacts could both be present, leading to different combinations of caregiving strain (along similar lines to recent developments in the study of professional burnout, see Leiter and Maslach, 2016). From this perspective, future research could consider personal accomplishment as a dimension that may counterbalance the other two negative dimensions and lead to different outcomes.

Future studies should also investigate the conceptual proximity of informal caregiver burnout to comparable concepts, in particular, compassion fatigue or satisfaction. Compassion fatigue is often referred as “the [professional] caregiver’s cost of caring” (Sorenson et al., 2016, p. 457) and compassion satisfaction “reflects the positive feelings that result from one’s ability to help others” (Lynch, 2018, p. 9). Some studies have adapted the concept of compassion satisfaction to the informal caregiving context as a measure of caregiving impact (e.g., Day et al., 2014; Lynch et al., 2018). Their results suggest that it is relevant to use it in informal care, but its overlap with informal caregiver burnout should be clarified. In occupational health research, this overlap between the two concepts is still being discussed, although compassion fatigue is often seen as a precursor of burnout (Sorenson et al., 2016). Theoretically, compassion fatigue seems to be close to emotional exhaustion and compassion satisfaction to personal achievement, but this proximity should be examined in future work.

#### General Outcomes

Beyond the specific impact of caregiving, more general outcomes can occur in reaction to or as a result of caregiver burnout. General outcomes of the caregiving impact are twofold: on the caregivers themselves and on the care-recipients. For the caregiver, informal caregiver burnout could lead to physical and psychological issues. In general, caregiver burnout is related to lower well-being, more psychological distress (Götze et al., 2015; Kindt et al., 2015; Bachner, 2016), more negative and less positive emotions (Kindt et al., 2015), and lower quality of life (Ostlund et al., 2010; Takai et al., 2011). In terms of psychopathology, some studies have highlighted a moderate relationship between burnout and anxiety (Yılmaz et al., 2009; Truzzi et al., 2012; Riva et al., 2014). As pointed out in the literature on professional burnout and on caregiving burden, the direction of these relationships is difficult to settle (Adelman et al., 2014; Chiao et al., 2015). Anxiety could be a consequence of burnout and the caregiver’s exhaustion, but trait anxiety could also lead to higher vigilance and overcaring, thus facilitating the occurrence of burnout. There is also a strong relationship between burnout and depression (Truzzi et al., 2008, 2012, p. 012; Yılmaz et al., 2009; Lee and Singh, 2010; Katsifaraki and Wood, 2014; Bachner, 2016). Caregiver burnout could also have an impact on other spheres, for example by putting the individual at risk of professional burnout (as shown for parental burnout in Greaves et al., 2017). Informal caregiver burnout could also be a key mediator between subjective burden and decreased social activity (Adelman et al., 2014): caregivers experiencing burnout are less likely to seek social contact. In terms of physical health, caregiver burnout is related to poorer subjective health (Valente et al., 2011; Choi and Sok, 2012; Goetzmann et al., 2012), and to more reported somatic symptoms (Weiss, 2002; Truzzi et al., 2012). Future research should further investigate the role of caregiver burnout in the erosion of the caregiver’s physical health and the mechanisms by which this impact occurs (e.g., health behaviors, psychoneuroendocrinology).

Informal caregiver burnout could have a direct impact on the well-being of the care-recipient (Kindt et al., 2015). Beyond that, caregiving strain could have an indirect impact through the onset of mistreatment (Wiglesworth et al., 2010; Fang and Yan, 2016). Despite the affective bond, the risk of neglect and abuse remains a reality (Acierno et al., 2010). Caregiver stress appears to be an important risk factor for the occurrence of physical or verbal violence (Johannesen and LoGiudice, 2013). Early results have shown a link between burnout and violence, in both formal (Truchot et al., 2013) and informal contexts (Yan, 2014).

Preliminary results highlight a potential relationship between the caregiver’s mental health and (re)admission rates of the patient (Longacre et al., 2014) as well as the likelihood of placement in nursing homes (Covinsky et al., 2003). No study has directly examined the impact of burnout on institutionalization. Similar to “turnover intention” in occupation contexts, a study has shown that caregivers of individuals with Alzheimer’s disease who stated that they would prefer their care-recipient to be in a nursing home reported higher burnout scores than those preferring to keep the care-recipient at home (Yılmaz et al., 2009). Future studies should thus expand these results to add to our understanding of the potential consequences of informal caregiver burnout on the care-recipient, and, to a larger extent, how these consequences affect the health care system.

#### Circularity

Beyond the consequences themselves, caregiver burnout and more general outcomes will in turn have an impact through feedback loops and modify elements regarding the caregiver, the caregiving context, and the social environment. This circularity has often been neglected in models created to understand caregiving strain (as well as in the JD-R model), but it is critical in addressing how caregiving strain may evolve. The modification of one element will have a global impact on caregiving strain through a direct and indirect modification of the caregiving experience. Longitudinal studies should thus investigate these loops and understand their pathways.

## Conclusion

The present work has proposed to adapt the concept of burnout to the informal caregiving context. This adaptation represents a response to criticisms made regarding the measurement of the impact of caregiving and its conceptual heterogeneity. The three-dimensional approach also expands the view of the impact of caregiving to the accomplishment found in the role and to the depersonalization that may occur. Additional studies should confirm the relevance of this concept, but it appears promising in light of the existing literature.

The second goal of the present work was to propose a theoretical model to frame future research on informal caregiver burnout. The review of the Job Demands-Resources Model from the occupation burnout literature and of the Caregiving Stress and Burden Model led to the development of the Informal Caregiving Integrative Model. This model aims to respond to the opportunities for the improvement of existing models, but also to preserve their most valuable features. The ICIM stresses the importance of taking full account of the actors and elements at stake when considering the determinants of caregiver burnout: the caregiving setting, the caregiver’s characteristics, and their sociocultural environment. Key mediators between the determinants and caregiver burnout are the caregiving appraisal (both positive and negative), and also the relationship between the care-recipient and the caregiver, which has often been neglected.

In adapting the burnout concept and providing an integrative model to address caregiver burnout, our purpose was not to provide an exhaustive treatment of this topic, nor to pretend to disprove existing research. Rather, the objective was to respond to critical work pointing to the need to re-explore informal caregiving research (Bastawrous, 2013; Mosquera et al., 2016). In the same way as other promising works (e.g., Revenson et al., 2016), the present article thus proposes new ideas for informal caregiving research, both in terms of impact measures and in terms of a conceptual framework for studying them.

## Author Contributions

PG and EZ contributed to the conception of this theoretical work. PG performed the literature review and wrote the first draft of the manuscript. EZ critically revised it. PG and EZ refined the manuscript for submission. This final version was approved by both authors.

### Conflict of Interest Statement

The authors declare that the research was conducted in the absence of any commercial or financial relationships that could be construed as a potential conflict of interest.
